# Reply: CIITA methylation and decreased levels of HLA-DR in tumour progression

**DOI:** 10.1038/sj.bjc.6602047

**Published:** 2004-07-27

**Authors:** Y Morimoto, M Toyota, H Ikeda, T Tokino, K Imai

**Affiliations:** 1 First Department of Internal Medicine, Sapporo Medical University, S-1, W-16, Chuo-ku Sapporo 060-8543, Japan

**Sir**,

We read with interest the comments and suggestions of Iizuka and Oka regarding our recent report on CIITA methylation in haematopoietic tumour cells (Morimoto *et al*, 2004). Using DNA microarray analysis of gene expression in hepatocellular carcinoma cells, which was followed by a combinational study using a supervised learning system and statistical analysis, Iizuka *et al* (2003) were able to isolate 12 genes that appear responsible for early intrahepatic recurrence of cancer after curative resection. One of those genes encoded HLA-DR alpha chain, whose expression was reduced significantly in hepatocellular carcinoma. In a subsequent study, however, they concluded that the respective expression levels of HLA-DR and CIITA were unrelated.

*CIITA* was originally isolated from a cDNA that restored expression of class II molecules on EBV-B lymphocytes derived from a patient with bare lymphocytes syndrome (Steimle *et al*, 1993). It is now generally accepted that CIITA is a master activator that regulates class II molecules as well as such class II-associated molecules as invariant chains and HLA-DM (Ting and Trowsdale, 2002). Four distinct promoter units are involved in the transcriptional regulation of CIITA, each transcribing a unique first exon, which is spliced to a common second exon (Muhlethaler-Mottet *et al*, 1997). Promoters I and III are used for the constitutive expression of CIITA in dendritic cells and B lymphocytes, respectively; the function of promoter II is unknown; and promoter IV controls IFN-*γ*-inducible CIITA expression in a variety of cell types. CIITA does not directly bind to MHC class II promoter regions containing cis-acting promoter elements (e.g. W-, X1-, X2- and Y-elements), but appears to exert its effects by trans-activation through protein/protein interactions with components of multiprotein complexes comprising the regulatory factor X family, which in turn activate transcription factor/cAMP-responsive element binding protein and NF-Y (Ting and Trowsdale, 2002). In a series of studies using knockout mice, CIITA was shown to be essential for the expression of class II molecules and T-cell mediated antigen responses (Chang *et al*, 1996; Williams *et al*, 1998). Indeed, transfection of CIITA cDNA into class-II negative cells restores cell surface expression of class II molecules, regardless of cell type (Kanaseki *et al*, 2003; Morimoto *et al*, 2004; Takamura *et al*, 2004). Consequently, at the present time there is no indication of a discrepancy between the expression of CIITA and that of class II molecules.

Whether comprising solid tumours or haematological malignancies, tumour cells can be classified into three categories based on their expression of class II molecules: (1) those that constitutively express class II molecules even without IFN-*γ*; (2) those that will express class II molecules with IFN-*γ*; and (3) those that will not express class II molecules even with IFN-*γ*. Several groups have investigated the relation between the expression of class II molecules and CIITA in cancers, and their findings indicate that cell type (1) is largely limited to melanoma and glioma (Deffrennes *et al*, 2001; Takamura *et al*, 2004) and that most other cancer cells would be classified as either type (2) or (3) (Liu *et al*, 1999; Soos *et al*, 2001; Yazawa *et al*, 2002).

Mechanisms for the inactivation of CIITA are much more complicated, however. Hypermethylation may be a key mechanism by which CIITA promoter activity is silenced in several cancers, as well as in trophoblasts under physiological conditions (van den Elsen *et al*, 2000; van der Stoep *et al*, 2002). In addition, we have reported that histone deacetylation, but not hypermethylation, modifies class II transactivator and MHC class II gene expression in squamous cell carcinomas (Kanaseki *et al*, 2003). In the same vein, we also reported the inactivation of class II transactivation by DNA methylation and histone deacetylation in T-cell and myeloid leukaemia cell lines (Morimoto *et al*, 2004), and that global histone acetylation in a region 6 kb upstream of transcriptional start site of CIITA promoter III in glioma cell lines leads to constitutive expression of class II molecules (Takamura *et al*, 2004). Thus, epigenetic alteration of different CIITA promoters may profoundly influence class II expression in different tissue types. We have not studied hepatocellular carcinomas; however, Sartoris *et al* (1998) reported successful induction of class II molecules after transfection of CIITA cDNA into a class II negative hepatocellular carcinoma cell line, which suggests that hepatocellular carcinoma may not be exceptional.

As Iizuka and Oka correctly pointed out, we did not investigate the relation between levels of CIITA and HLA-DR and tumour progression. They proposed that we extend our study to relate CIITA methylation with the expression of class II molecules in a larger cohort of haematopoietic tumours. We like this idea, although it clearly goes beyond the primary scope of our investigation.

Finally, we noted with interest the findings of Hippo *et al*, who analysed global gene expression as Iizuka and Oka did. They compared murine scirrhous gastric cancer cells with their highly metastatic derivatives in lymph nodes using the high-density oligonucleotide array method. This enabled them to isolate a cluster of upregulated genes, including MHC class II genes, which were induced via CIITA (Hippo *et al*, 2001), suggesting that CIITA upregulates class II expression during tumour progression. Moreover, the fact that melanoma and glioma constitutively express class II molecules via CIITA should not be ignored. We thus need to be careful not to draw a simple picture in which only inactivation of CIITA or class II molecules leads to escape from immune surveillance and a poor prognosis.
